# Broad-Spectrum Detection of HPV in Male Genital Samples Using Target-Enriched Whole-Genome Sequencing

**DOI:** 10.3390/v15091967

**Published:** 2023-09-21

**Authors:** Tengguo Li, Elizabeth R. Unger, Mangalathu S. Rajeevan

**Affiliations:** Division of High-Consequence Pathogens & Pathology, Centers for Disease Control and Prevention, 1600 Clifton Road, Atlanta, GA 30329, USA; uyy7@cdc.gov (T.L.); eru0@cdc.gov (E.R.U.)

**Keywords:** universal HPV typing assay, mixed infection (alpha; beta; gamma types), male genital samples, epithelial tropisms, vaccines

## Abstract

Most human papillomavirus (HPV) surveillance studies target 30–50 of the more than 200 known types. We applied our recently described enriched whole-genome sequencing (eWGS) assay to demonstrate the impact of detecting all known and novel HPV types in male genital samples (*n* = 50). HPV was detected in nearly all (82%) samples, (mean number of types/samples 13.6; range 1–85), and nearly all HPV-positive samples included types in multiple genera (88%). A total of 560 HPV detections (237 unique HPV types: 46 alpha, 55 beta, 135 gamma, and 1 mu types) were made. The most frequently detected HPV types were alpha (HPV90, 43, and 74), beta (HPV115, 195, and 120), and gamma (HPV134, mSD2, and HPV50). High-risk alpha types (HPV16, 18, 31, 39, 52, and 58) were not common. A novel gamma type was identified (now officially HPV229) along with 90 unclassified types. This pilot study demonstrates the utility of the eWGS assay for broad-spectrum type detection and suggests a significantly higher type diversity in males compared to females that warrants further study.

## 1. Introduction

Human papillomaviruses (HPV) are DNA viruses in the family *Papillomaviridae* that includes 49 genera. More than 200 HPV types that infect cutaneous and mucosal surfaces have been found in five of these genera, with most in alpha, beta, and gamma genera. While most infections clear, persistent infection with some alpha types (HPV16, 18, 31, 33, 35, 39, 45, 51, 52, 56, 58, 59, 66, and 68) is a risk factor for anogenital and oropharyngeal cancers [1]. Beta and gamma types have not been studied systematically. However, recent reports have detected alpha, beta, and gamma types in oral samples [2], cervical cancers [3], head and neck cancers [4], anal and genital areas of males [5], and juvenile-onset recurrent respiratory papillomatosis (JoRRP) [6]. In addition, interactions between HPV types in different genera may have biologic and clinical significance, as reported for the differential interaction of HPV16 with beta and gamma HPVs in head and neck cancers [7], the association of alpha and beta HPV coinfection with the clinical severity of JoRRP [6], and the detection of a high diversity of alpha, beta, and gamma HPVs in penile samples from males infected with HIV [8]. Mechanistic studies suggest that some beta 3 types, such as HPV49, 75, and 76, can immortalize primary keratinocytes such as those immortalized with high-risk alpha HPV16 [9]. Beta types may act as co-carcinogens with UV radiation for nonmelanoma skin cancers (NMSC) [10]. Individuals with epidermodysplasia verruciformis (EV), a rare autosomal recessive hereditary skin disorder, have a high susceptibility to beta HPV infection and NMSC [11]. Gamma HPVs can induce cutaneous lesions, and gamma 11 and 12 species were recently shown to be associated with head and neck squamous cell carcinoma (HNSCC) [4]. The widespread occurrence of alpha, beta, and gamma HPVs in various anatomical sites indicates the importance of studies to clarify their natural history and pathogenesis, and the potential impact of HPV vaccines on their prevalence.

Currently, most studies have used PCR assays with degenerate primers (FAP59/64, BGC-PCR, and CUT primers) to detect and type HPV within specific genera [12,13]. Epidemiological studies in men frequently use self-collected external genital swabs that sample both mucosal and cutaneous epithelia, yet most HPV typing assays only target mucosal types that are mainly alpha types [14]. Next-generation sequencing (NGS)-based assays are making advances with viral genome research, and a few NGS studies, such as FAP PCR followed by 454 sequencing or Illumina MiSeq, report detection of alpha, beta, and gamma types using amplicons of limited sizes [8,15,16]. NGS following rolling-circle amplification (NGS-RCA) has also been reported with moderate success [17]. There are no NGS-based reports of detecting multiple HPV types belonging to different genera from any anatomical site in a single reaction. We recently developed a universal HPV assay based on target-enriched whole-genome sequencing (eWGS) to detect and type all known and potentially novel HPVs in a single reaction [18]. Anticipating that samples from keratinized (cutaneous) and non-keratinized (mucosal) surfaces of male genital tract would have high HPV diversity, we used eWGS to detect and identify HPV in a pilot study of 50 male genital samples. 

## 2. Materials and Methods

### 2.1. Samples

We used residual anonymized DNA extracts (*n* = 50) from external genital swabs in specimen transport medium collected from US males between 2010 and 2011 (IRB determination—Exempt). The vaccination history was unknown, but collection was prior to the US recommendation of routine HPV vaccination for males. DNA extracts were prepared using a Chemagic gDNA kit with an automated Chemagic MSM1 extractor (Perkin Elmer, Waltham, MA, USA) and stored at −80 °C for 63–68 months prior to use in the eWGS assay. The DNA concentration of the thawed extracts was determined using a Qubit dsDNA HS assay (Life Technologies, Eugene, OR, USA).

### 2.2. HPV Typing by Enriched Whole-Genome Sequencing (eWGS) Assay

The eWGS assay was conducted as previously described [18] with HPV genome enrichment using RNA probes (also referred to as baits) covering the full length of 202 HPV types (183 officially classified and 19 unclassified). A total of 13 controls were included. Water and human placenta DNA (Sigma-Aldrich, St Louis, MO, USA) were prepared in duplicate as HPV-negative controls. The HPV-positive controls included SiHa cell line DNA (HPV16 positive, 10 ng/reaction in duplicate) and 7 pools of HPV plasmids representing a total of 18 HPV types; 10 alpha types (HPV6, 11, 16, 18, 31, 33, 45, 52, 53, and 58), 7 beta types (HPV5, 8, 15, 20, 23, 24, and 36), and 1 gamma type (HPV48). Beta and gamma HPV plasmids (all full length except HPV5b and HPV24) were obtained from Karolinska Institute (Stockholm, Sweden). HPV5b was cloned into two plasmids with insert sizes designated as 5–5.8 kb and 5–1.9 kb. HPV24 was also cloned into two plasmids with fragments designated as 24–5.1 kb and 24–1.8 kb. Plasmids containing the full-length genomes of 9 vaccine HPV types (HPV6, 11, 16, 18, 31, 33, 45, 52, and 58) and HPV53 were received from various sources including the American Type Culture Collection (Manassas, VA, USA), German Cancer Research Institute (Heidelberg, Germany), Karolinska Institute (Stockholm, Sweden), and Institute Pasteur (Paris, France). All controls were tested in duplicate, and within each plasmid pool, the individual types were present at the same copy number (625 copies/reaction).

Due to variation in the input DNA (10–100 ng/sample; mean ± SD = 27.3 ± 23.6), HPV-negative placental DNA was added to bring the amount of DNA/sample to 100 ng prior to shearing (Supplemental Table S1). Libraries from each of the 63 samples (13 controls and 50 extracts) were prepared and barcoded as previously described [18]. The barcoded samples were pooled (15–16 per pool) at equal molar amounts (pre-capture pooling) and enriched through hybridization (65 °C for 24 h) with HPV bait. Each pool contained at least one HPV-negative and one -positive control. The pooled captured libraries were amplified using 14 cycles of PCR, followed by purification with Ampure beads (Beckman Coulter, Indianapolis, IN). Quantity and quality of the captured libraries were assessed by a Bioanalyzer 2100 (Agilent Technologies, Santa Clara, CA, USA) and qPCR using the Kapa library quantification kit (Roche, Indianapolis, IN, USA) on a Light Cycler 480 (Roche Diagnostics, Indianapolis, IN, USA). The four target enriched libraries were combined at equal molar concentrations to make 2 sequencing libraries that were paired end sequenced at 3.2 pM on a two-lane flow cell using a Illumina HiSeq Rapid SBS kit (200 cycles) on a Hiseq 2500 (Illumina, San Diego, CA, USA). 

### 2.3. Bioinformatics 

The sequence data were analyzed using CLC Genomics Workbench (CLC Bio, Waltham, MA, USA). Read de-multiplexing, quality control, alignment, and HPV type determination were performed as described previously [18], except that reads were mapped at 90% similarity to 445 reference genomes (including both classified and unclassified types) available at PAVE (https://pave.niaid.nih.gov/) (as of 24 February 2022). A sample was considered positive for an HPV type if there were at least 100 mapped reads that covered at least 20% of a classified or unclassified reference HPV genome. This cut off is more stringent than recommended (minimum 10 reads and 10% genome coverage) for determining positive signal in HPV whole-genome studies [19].

## 3. Results

Sequencing libraries 1 (samples 1–31) and 2 (samples 32–63) generated similar cluster densities of 962 K/mm^2^ and 995 K/mm^2^, with a total of 30, 935, and 33,083 mega base (Mb) sequence data, respectively. All samples with DNA generated an average of 10.5 million reads/sample with a mean Q score of 36.2; 93.4% of the bases had a Q score ≥ 30. Results from analysis of these data are presented below. The 13 control samples gave the expected results (Supplementary Table S2). 

### 3.1. HPV Identification in Male Genital Samples

#### 3.1.1. Read and Mapping Characteristics

Of 50 male genital samples, 41 (82%) were HPV eWGS-positive. There were 560 total HPV detections of two-hundred thirty-seven unique HPV types in four genera: forty-six alpha, fifty-five beta, one-hundred thirty-five gamma, and one mu types. Within genera, the types were spread across forty-two species: fourteen alpha, five beta, and twenty-three gamma species. The number of HPV types per sample ranged from 1 to 85 (mean = 13.6 types per sample). Of the 560 HPVs detected, 72% (402) were officially classified and 28% (158) were unclassified in the PAVE database. Gamma HPV detections dominated (46.3%, 257/560), followed by beta (32%, 179/560), alpha (22.8%, 122/560), and mu genomes (0.35%, 2/560). 

Among the 122 alpha HPV detections, 46 unique types were identified in 32 samples. The number of reads/alpha genome was 88,996 ± 29,619 and >80% of the genome was sequenced in over 75% (94/122) of these detections. Among the 179 beta HPV detections, 55 unique beta types were identified in 35 samples. The number of reads/beta genome was 17,020 ± 4769, and >80% of the genome was sequenced in over 57% (102/179) of the detections. Among the 257 gamma HPV detections, 135 unique types were identified in 36 samples. The number of reads/gamma genome was 36,585 ± 9738, and >80% of the genome was sequenced in nearly 42% (107/257) of the detections. The two mu papillomavirus signals were mapped from two samples (reads/genome = 529 ± 444, genome coverage = 0.3 ± 0.12) to the same unclassified novel type, HPV-md01c06. 

**Figure 1:**
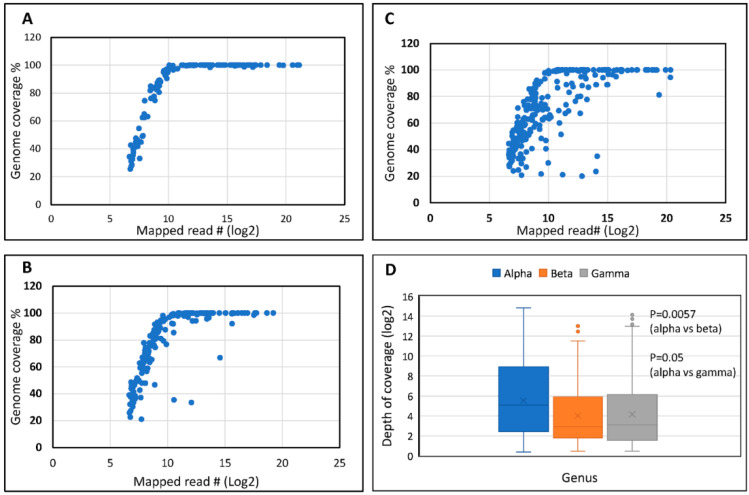
Mapped Reads and Genome Coverage. Correlation shown for types by genus: (**A**) alpha, (**B**) beta, and (**C**) gamma. (**D**): Boxplot of depth of coverage by genus (log 2 scale; antilog of 10 to the base 2 = 2^10^ = 1024).

The relationship between the number of reads and the genome coverage followed the expected linear relationship until coverage reached 100% (Figure 1A–C). The full range of mapped reads was also correlated (r^2^ = 1) with the depth of coverage (Supplementary Figure S1A), as expected. On average, 400 ± 183 reads covered approximately 60% of the genome, and 95–100% of the HPV genomes were covered fully by approximately 1500 ± 200 reads. Alpha genomes had the highest number of reads, and the depth of coverage (mean ± SD = 1121 ± 373.4) was significantly higher (Figure 1D) compared to that of beta (227.96 ± 74.46; *p* = 0.0057) and gamma genomes (502.63 ± 133.55; *p* = 0.05). The GC content (%) was significantly higher in alpha genomes (range 36.24–48.63%; mean ± SD = 41.57 ± 3.51) compared to both beta (range 36.30–45.52%; mean ± SD = 40.63 ± 1.24; *p* = 0.001) and gamma genomes (range 35.57–46.67%; mean ± SD = 37.97 ± 1.71; *p* ≤ 0.0001) (Supplementary Figure S1B).

#### 3.1.2. Broad Spectrum of HPV Genomes in Male Genital Samples

**Figure 2:**
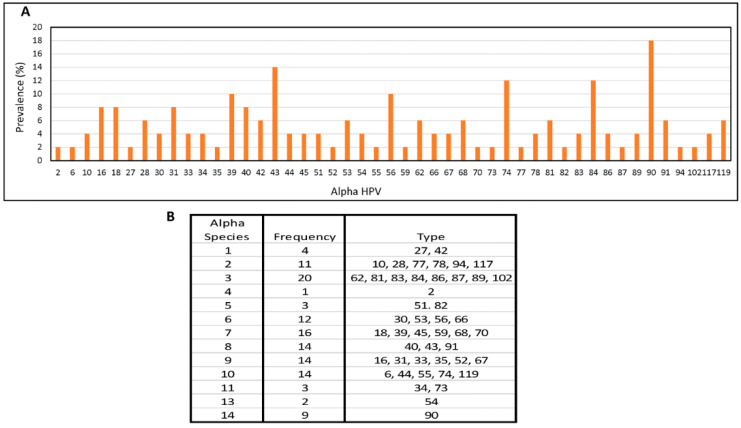
Alpha HPV in Male Samples. Prevalence by type (**A**) and species (**B**). All alpha types detected were officially classified.

The number of alpha types detected per sample ranged from 1 to 21 (mean = 4/sample). While the most common alpha types were HPV90, HPV43, HPV74, and HPV84 (Figure 2A), the greatest number of reads were seen with types 91 (2,303,350 reads) and type 39 (207,750 reads). All alpha types detected were officially classified. Except for HPV58, all types (HPV16, 18, 31, 33, 35, 39, 45, 51, 52, 56, 59, and 66) classified as Group 1 carcinogens by the International Agency for Research on Cancer (IARC) were detected a total of 33 times. At species level, alpha 3 types (HPV 62, 81, 83, 84, 86, 87, 89, and 102) were detected twenty times, alpha 7 (HPV18, 39, 45, 59, 68, and 70) were detected sixteen times, and alpha 8 (HPV40 and 43), alpha 9 (HPV16, 31, 33, 35, 52, and 67), and alpha 10 (HPV6, 44, 55, 74, and 119) were each detected eight times (Figure 2B). 

**Figure 3:**
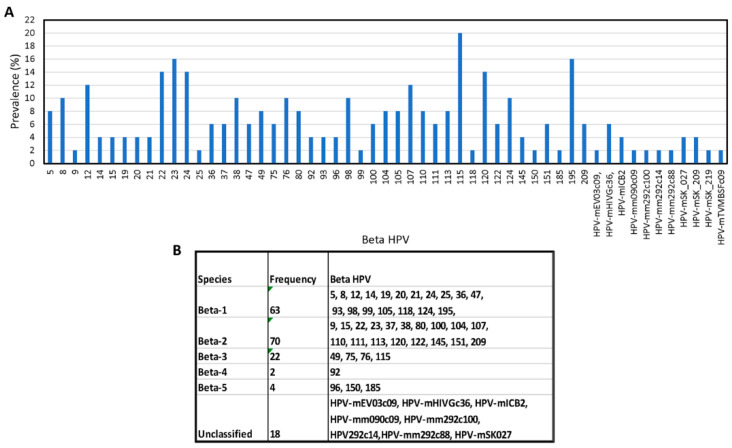
Beta HPV in Male Samples. Prevalence by type (**A**) and species (**B**). A total of 55 unique beta types were detected that included 11 unclassified types.

The number of beta types detected per sample ranged from 1 to 26 (mean = 5/sample). While the most common beta types were HPV types 115, 195, 120, 22, 23, 24, and 120 (Figure 3A), the greatest number of reads were seen with type 23 (604,935 reads) and type 22 (413,672 reads). Among the 55 unique beta types, 44 were officially classified and 11 were unclassified. At species level, beta 1 types (HPV5, 8, 12, 14, 19, 20, 21, 24, 25, 36, 47, 93, 98, 99, 105, 118, 124, and 195) were detected 63 times, beta 2 (HPV9, 15, 22, 23, 37, 38, 80, 100, 104, 107, 110, 111, 113, 120, 122, 145, 151, and 209) 70 times, and beta 3 (HPV49, 75, 76, and 115) 22 times (Figure 3B). 

**Figure 4:**
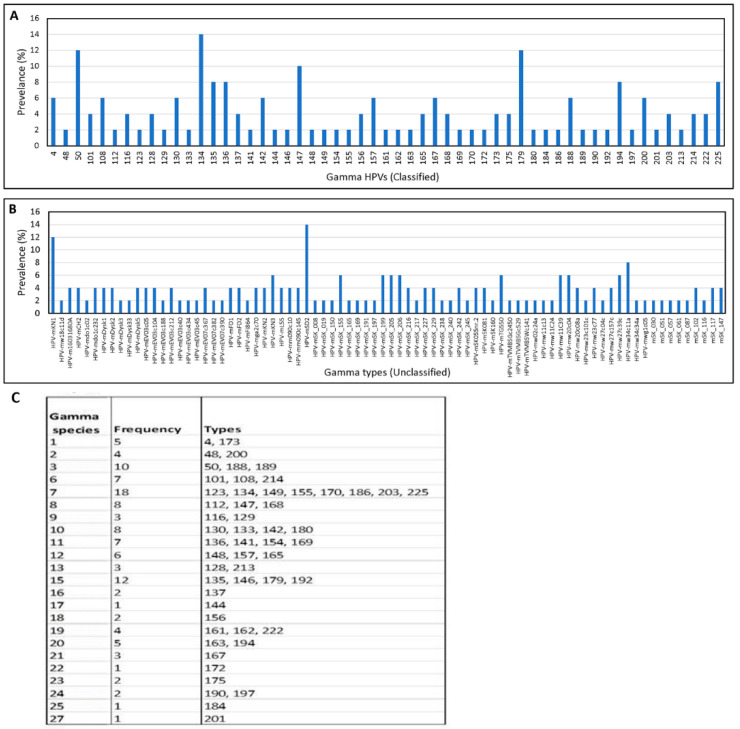
Gamma HPV in Male Samples. Prevalence by type ((**A**) 55 types classified, (**B**) 80 types unclassified) and species (**C**).

The number of gamma HPV types detected per sample ranged from 1 to 53 (mean = 7/sample). While the most common gamma types were HPV134, HPVmSD2, HPV 50, HPV179, HPVmKN1, and HPV147 (Figure 4A,B), the greatest number of reads were seen with HPV50 (1,322,570 reads), HPV mDySK3 (1,320,464 reads), and HPV184 (990,359 reads), respectively. Among the 135 unique gamma HPV types, 55 were officially classified and 80 were unclassified. Only types with official classification are assigned to species. Among the 23 gamma species, the most prevalent were gamma 7 types (HPV123, 134, 149, 155, 170, 186, 203, and 225) which were detected eighteen times, gamma 15 (HPV135, 146, 179, and 192) which were detected twelve times, gamma 3 (HPV50, 188, and 189) which were detected eight times, and gamma 6 (101, 103, and 214) which were detected seven times (Figure 4C).

**Figure 5:**
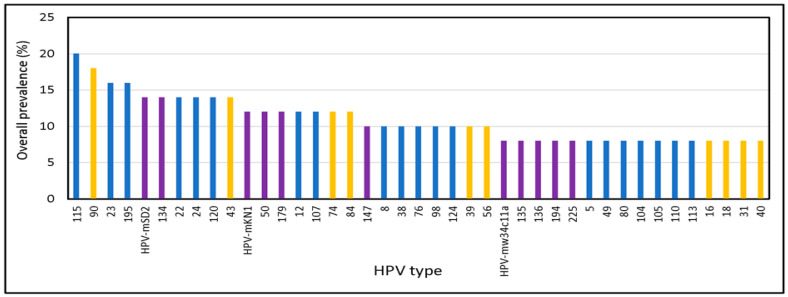
Overall HPV Prevalence in Male Samples. Alpha, beta, and gamma types are indicated by orange, blue, and purple bars, respectively.

The overall prevalence of male genital HPVs belonging to alpha, beta, and gamma genera is presented in Figure 5. Three of the four most prevalent types were gamma types, and gamma genomes dominated in 16 samples, followed by beta (12 samples) and alpha genomes (6 samples). Multi-genera detection was much more common (88% of HPV-positive samples) than single genus detection (12%), which was seen in only 5 samples (three samples with beta HPVs and two samples with gamma HPVs, Supplemental Table S3). Co-detection of alpha, beta, and gamma genomes occurred in 25 samples, and co-detection of pairs of alpha, beta, and gamma genomes occurred in 11 samples.

### 3.2. Identification of Novel and Unclassified Types 

Interestingly, of the 237 unique HPV types identified by eWGS, there were no RNA baits in the enrichment hybridization for ninety-seven types (fourteen beta, eighty-two gamma, and one mu types) and eighty-three of these types were unclassified. The study also identified a novel HPV genome (GenBank Accession # MW535770), that has subsequently been officially classified by the International HPV Reference Center, Sweden, as gamma HPV type 229.

## 4. Discussion

**Figure 6:**
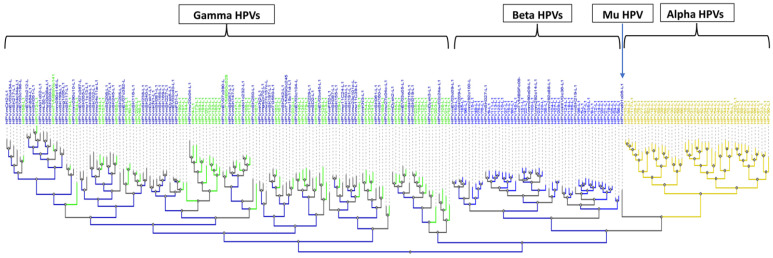
Phylogenetic Tree of HPVs Detected in Male Samples. The tree is based on the L1 sequence of HPVs detected in this study, constructed using the tools at the PAVE EPSTEIME database. HPVs are grouped within alpha, beta, gamma, and mu (in green are gamma types with official classification). Tree branches in gray under gamma and beta indicate unclassified types. No unclassified types under alpha.

This pilot study was designed to demonstrate the capacity of the eWGS to identify all known and unknown HPVs in samples from male external genital swabs that include both mucosal and cutaneous epithelial surfaces. The expected universal capacity of the eWGS assay to detect HPV was supported by the detection of two-hundred thirty-seven unique types from four of the five *Papillomaviridae* genera recognized to have HPV types (forty-six alpha, fifty-five beta, one-hundred thirty-five gamma, and one mu types) (Figure 6). For three of the genera, multiple species were detected (fourteen species of alpha, five species of beta and twenty-three species of gamma). The broad typing capacity of the assay resulted in a high number of multiple types detected per sample (mean 13.6, range 1–85). This extensive diversity and high prevalence of HPV in male genital samples suggests further studies are warranted to evaluate the potential for males to serve as a reservoir for a large number of HPVs.

It is interesting to note that of the five most prevalent high-risk types in a meta-analysis of data from women with normal cervical cytology in the pre-vaccine era (HPV16, 18, 31, 52, and 58) [20], HPV 58 was not detected in the male samples in this study. Low-risk alpha types HPV90 (18%), 43 (14%), and 84 and 74 (each at 12%) were more prevalent than high-risk alpha types HPV16, 18, and 31 (each at 8%) in our study. Alpha 3 types (HPV62, 84, and 89) were the most prevalent low-risk types in male genital samples in the National Health and Nutrition Examination Survey of adult US men [21] and women [22]. A higher prevalence of HPV84 than of HPV16 has been reported in prior studies of men from culturally and geographically diverse regions of the world [23]. Also, in agreement with that report, we did not identify HPV 61, a common vaginal type that is also an alpha 3 species like HPV 84 [24].

Alpha types, the major genus detected in cervicovaginal samples, were not the most frequently detected in male genital samples, although they were present at a higher copy number (averaging two to five times more reads/sample than beta and gamma genomes) and had the highest percentage (75%, 94/122) of complete genomes. It is also striking that the HPV diversity in male genital samples in our study was greater than that reported in cervical samples tested with the same eWGS assay [18,25]. Prior studies of cervicovaginal samples using broad-spectrum primer systems (FAP 59/64 and MY09/11 followed with [3] or without [26] NGS, gamma PCR, and CUT PCR [27]) also did not demonstrate the broad representation of HPV genera we report in our male samples. This may be due to the combination of mucosal and cutaneous surfaces in males that present unique environmental niches supporting HPV type diversity as well as to differences in assay sensitivity for a very broad range of types. 

Prior studies of male genital samples using multiple assays (linear array followed by sequencing of PCR products of PGMY 09/11 and FAP59/64 primer systems) found fewer than 20 unique beta types largely in one beta species (beta 2) [28]. However, we detected 55 beta types representing all five beta species. All four beta 3 types 49, 75, 76, and 115 were detected in this study. We believe our study is the first to identify HPV 115, originally isolated from skin [13], as the most prevalent type in male genital samples (Figure 5A). 

We identified 55 of the 98 types within gamma, the genus with the largest number of types with official classification. Of the unclassified types detected in our study, the largest number (87%; 80/92) were in this genus. Among gamma types, the prevalence was highest for HPV-mSD2 and HPV 134, each at 14%, followed by HPV-mKN1, HPV 50, and HPV147, each at 12% in this study. HPV-mSD2 and HPV134 were previously identified as the most prevalent gamma types in oral rinse samples [29]. 

This pilot study has several limitations. It used residual samples without demographic information, and results cannot be considered representative of any population. Storage may have compromised sample quality, although quality control suggested this was not an issue. HPV vaccination status was unknown, although the dates of the original sample collection were before widespread male vaccination. Enrichment methods, particularly those that rely on PCR, may bias the observed type distribution. As eWGS uses a limited number of PCR cycles and maintains a strong linear relationship between target copies and signal strength [30], this concern may be lessened.

This pilot study demonstrates the usefulness of eWGS for broad-spectrum detection of HPV genomes belonging to multiple genera. Systematic studies using eWGS will generate a more complete view of HPV type distribution, to better understand their biology, tissue tropism, and potentially new disease associations. 

## Data Availability

The data presented in this study are available on request from the corresponding author.

## Supplementary Materials

The following supporting information can be downloaded at: https://www.mdpi.com/article/10.3390/v15091967/s1, Figure S1: Linear relationship between number of reads and depth of coverage; Figure S2: GC content (%) of alpha, beta, and gamma genomes in male genitals. Table S1: Sample arrangement and DNA input for library preparations; Table S2: HPV type determination in controls, and Table S3: Multi-genera detection of HPV in male genitals identified by eWGS.
