# Supplementary material for: Broad-Spectrum Detection of HPV in Male Genital Samples Using Target-Enriched Whole-Genome Sequencing
Source: Viruses. 2023 Sep 21;15(9):1967. doi: 10.3390/v15091967 (PMC10538195; doi:10.3390/v15091967)
Supplement: Supplementary file 1 [file viruses-15-01967-s001.zip › viruses-2619251-supplementary.pdf]

Table S1: Sample arrangement and DNA input for library preparations

Sample No.	Specimen	DNA input/reaction (ng*)	Placental DNA (ng)	Capture library (#)	Sequencing library (#)
1	Genital swab	13.6	86.4		
2	Genital swab	19.5	80.5		
3	Genital swab	16.7	83.3		
4	Genital swab	23.6	76.4		
5	Genital swab	28.0	72.0		
6	Genital swab	14.7	85.3		
7	Genital swab	19.6	80.4		
8	Genital swab	100.0	0.0		
9	Genital swab	13.0	87.0	1	
10	Genital swab	15.8	84.2		
11	Genital swab	21.0	79.0		
12	Genital swab	30.6	69.4		
13	Genital swab	12.6	87.4		
14	Genital swab	18.4	81.6		
15	H_2O	0.0	0.0		
16	SiHa	10.0	90.5		1
17	Genital swab	13.7	86.3		
18	Genital swab	14.7	85.3		
19	Genital swab	28.1	71.9		
20	Genital swab	100.0	0.0		
21	Genital swab	11.1	88.9		
22	Genital swab	26.5	73.5		
23	Genital swab	52.5	47.5		
24	Genital swab	12.4	87.6	2	
25	Genital swab	17.9	82.1		
26	Genital swab	13.4	86.6		
27	Genital swab	19.5	80.5		
28	Genital swab	17.3	82.7		
29	Genital swab	33.2	66.8		
30	Placenta	100.0	100.5		
31	Plasmid pool (HPV11, 16, 31, 45, 52)	625 copies/plasmid	100.0		
32	Genital swab	12.8	87.2		
33	Genital swab	14.1	85.9		
34	Genital swab	19.4	80.6		
35	Genital swab	100.0	0.0		
36	Genital swab	22.8	77.2		
37	Genital swab	26.9	73.1		
38	Genital swab	18.3	81.7		
39	Genital swab	10.9	89.1		
40	Genital swab	17.8	82.2	3	
41	Genital swab	24.0	76.0		
42	Genital swab	12.5	87.5		
43	HPV plasmid pool (5,8,23,26)#	625 copies	100.0		
44	Plasmid pool (HPV6,16,20,24,36,58)&	625 copies	100.0		2
45	HPV plasmid pool (5,11,15,45,52)	625 copies	100.0		
46	H_2O	0.0	0.0		
47	SiHa	10.0	90.0		
48	Genital swab	28.7	71.3		
49	Genital swab	100.0	0.0		
50	Genital swab	15.1	84.9		
51	Genital swab	29.9	70.1		
52	Genital swab	28.4	71.6	4	
53	Genital swab	40.7	59.3		
54	Genital swab	12.2	87.8		
55	Genital swab	18.1	81.9		

56	Genital swab	34.0	66.0		
57	Genital swab	12.0	88.0		
58	Genital swab	49.7	50.3		
59	Genital swab	10.3	89.7		
60	HPV plasmid pool (15,20,24,48)	625 copies/plasmid	100.0		
61	HPV plasmid pool (8,18,23,31,33,48,53)	625 copies/plasmid	100.0		
62	Placenta	100.0	100.0		
63	HPV plasmid pool (6,33,18,53, 58)	625 copies/plasmid	100.0		

*Placental DNA was added to the indicated DNA input from genital swabs to make it 100 ng for the library preparation.

#HPV5 was cloned in 2 separate plasmids (1.9kb and 5.8kb, respectively)

& HPV24 was cloned in 2 separate plasmids (2.8 kb and 5.1kb, respectively)

Table S2. HPV type determination in controls

Sample No.	Sample description	Mapped reads	Fraction of reference covered	HPV Type call		
				eWGS	Expected	Concordance
15	H_2O			HPV-	HPV-	Yes
16	Siha	21995	0.95	16	16	Yes
30	Placenta			HPV-	HPV-	Yes
31	5 HPV plasmids (45,52,16,11,31)	4434	1.00	11	11,16,31,45,52	Yes
		6162	1.00	16		
		4306	1.00	31		
		6239	1.00	52		
		6494	0.99	45		
43	4 HPV plasmids (36,23,8,5-5.8kb,5-1.9kb)	3622	1.00	8	5,8,23,36	Yes
		3726	1.00	23		
		4051	1.00	36		
		3255	0.99	5		
44	7 HPV plasmids (6,16,24-5.1kb, 24-1.8kb,20,36,58)	6174	1.00	16	6,16,20,24,36,58	Yes
		4593	1.00	24		
		6246	1.00	58		
		3260	1.00	6		
		5424	1.00	36		
		4007	1.00	20		
45	6 HPV plasmids:5-5.8kb,5-1.9kb,11,15,45,52)	3373	1.00	15	5,11,15,45,52	Yes
		5504	1.00	52		
		4823	1.00	11		
		3704	0.99	5		
		6092	0.99	45		
46	H_2O			HPV-	HPV-	Yes
47	SiHa	23932	0.95	16	16	Yes
60		5020	1.00	24	15,20,24,48	Yes

	5 HPV plasmids (15,20,24-5.1kb, 24-1.8kb,48)	3913	1.00	15		
		4706	1.00	48		
		4385	0.99	20		
61	7 HPV plasmids (8,18,23,31,33,48,53)	6451	1.00	18	8,18,23,31,33,48,53	Yes
		4441	1.00	23		
		4448	1.00	31		
		5897	1.00	33		
		3898	1.00	48		
		7396	1.00	53		
		4197	1.00	8		
62	Placenta			HPV-	HPV-	Yes
63	5 plasmids (6,33,18,58,53)	7277	1.00	18	6,33,18,58,53	Yes
		7621	1.00	33		
		8993	1.00	53		
		7547	1.00	58		
		4102	1.00	6		

All controls were tested in duplicate, and within each plasmid pool, the individual types were present at the same copy number (625 copies/reaction). The 13 control samples gave the expected results : the SiHa cell line was positive for HPV16 and no reads from HPV negative controls were mapped to HPV genomes. The mean number of mapped reads/HPV type in the plasmid pool was 5160.8 ± 235. Further, all 18 HPV types in various plasmid pools were 100% concordant to the expected types in these pools.

Table S3. Multi-genera detection of HPV in male genitals identified by eWGS.

Sample#	Alpha	Beta	Gamma	Mu	Types#
3	4	5	1	0	10
4	0	4	1	0	5
5	4	1	0	0	5
7	2	4	14	0	20
8	1	4	2	0	7
9	1	1	0	0	2
10	0	0	2	1	3
11	5	1	4	1	11
12	7	7	5	0	19
17	0	0	1	0	1
19	3	4	25	0	32
20	2	1	1	0	4
21	7	6	19	0	32
22	11	25	49	0	85
23	0	9	5	0	14
26	0	0	1	0	1
27	0	3	1	0	4
28	0	16	14	0	30
29	0	4	10	0	14

30	3	3	7	0	13
33	0	4	1	0	5
34	4	2	6	0	12
35	6	1	3	0	10
37	1	11	4	0	16
38	0	6	0	0	6
39	1	2	1	0	4
40	4	5	11	0	20
41	2	1	2	0	5
42	1	5	5	0	11
43	0	5	0	0	5
49	1	1	2	0	4
50	2	0	8	0	10
51	3	4	9	0	16
52	0	2	0	0	2
53	2	1	1	0	4
54	3	0	6	0	9
55	21	21	20	0	62
56	4	3	8	0	15
57	5	2	3	0	10
58	5	5	2	0	12
59	7	0	3	0	10
Total	122	179	257	2	560

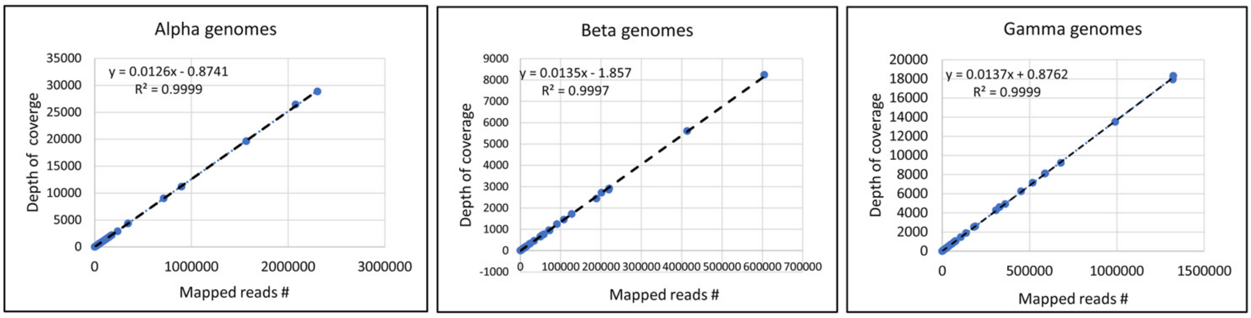

Figure S1: Linear relationship between number of reads and depth of coverage.

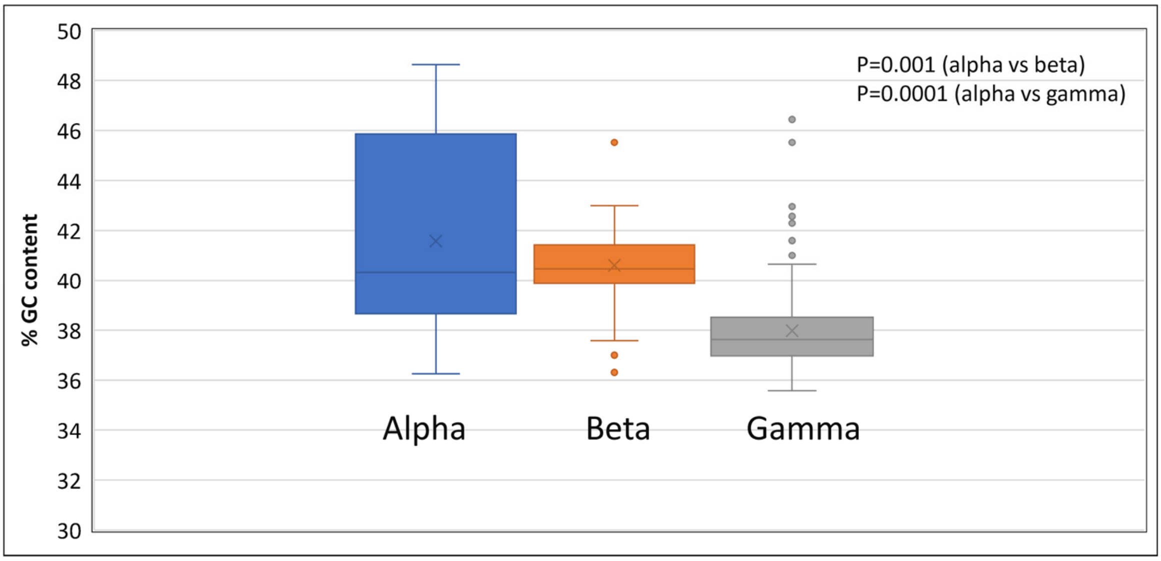

Figure S2: GC content (%) of alpha, beta, and gamma genomes in male genitals.
